# The power of metaphor in medical education: fostering shared understanding in complex conversations

**DOI:** 10.3389/fmed.2025.1703741

**Published:** 2025-12-10

**Authors:** Aaron Johnston, Sandra Andrawis, Grace Perez, Veronique Ram, Samuel Ogbeide

**Affiliations:** 1 Distributed Learning and Rural Initiatives, Cumming School of Medicine, University of Calgary, Calgary, AB, Canada; 2 Department of Emergency Medicine, Cumming School of Medicine, University of Calgary, Calgary, AB, Canada; 3 Department of Family Medicine, Cumming School of Medicine, University of Calgary, Calgary, AB, Canada; 4 Erin Ridge Family Health Centre, St. Albert, AB, Canada; 5 Riverside Medical, Drumheller, AB, Canada; 6 Department of Family Medicine, University of Alberta, Edmonton, AB, Canada

**Keywords:** medical education, undergraduate medical education, clinical reasoning, communication, difficult conversations, metaphor, neurodivergent, neurotypical

## Abstract

Effective communication in medical education, particularly around complex or emotionally charged topics, remains a significant challenge. While numerous feedback frameworks exist, less attention has been paid to optimizing fundamental linguistic tools like metaphor. Contemporary theory posits metaphor not merely as linguistic ornamentation, but as a conceptual tool that can deepen and even create understanding. This article employs a conceptual review format, drawing on established principles from cognitive psychology, narrative medicine, and adult learning theory. It utilizes illustrative vignettes to present practical applications of metaphor in clinical training environments. We identify, and explore, three key areas where metaphor can be a powerful tool for medical educators: (i) Teaching clinical reasoning, (ii) Opening difficult conversations, (iii) Facilitating discussions on professional identity formation. We identify challenges with metaphor use, such as male gender coded metaphor, and explore areas of caution for the use of metaphor in medical education, including intercultural communication and communication with neurodiverse individuals. We use a strategy called SAFE (Slow down, Acknowledge and Apologize, Follow-up, Explain), as a simple mnemonic for educators to recognize and respond when a metaphor misses the mark. Metaphor is experiencing a renaissance in medical education as a vital tool for fostering shared understanding. Its intentional use can enhance teaching in complex domains like clinical reasoning, difficult conversations, and professional identity. Educators are encouraged to adopt this tool mindfully, with awareness of its potential pitfalls, and a readiness to employ the SAFE strategy when a miss is perceived.

## 1 Introduction

Effective communication in medical education remains a critical challenge, particularly around addressing complex concepts and difficult topics. Despite substantial faculty development efforts targeting feedback, communication challenges persist. Numerous frameworks for feedback and communication have been developed (1), but less attention has been given to the role of optimizing basic tools of language, such as metaphor, in medical education.

In primary school we learn that metaphors are a linguistic tool for comparison, but theoretical work on the topic tells us that metaphor can be much more. Reddy and Lakoff describe metaphor not as an issue of language, or how we speak, but as an issue of conception, meaning how we think (2–4). This contemporary theory of the conceptual metaphor asserts that metaphor has the power to deepen, and even to create understanding. The thoughtful use of metaphor offers an enticing tool to overcome some of the communication challenges physicians experience in day-to-day teaching (5). The power of metaphor in medical communication lies in its ability to transform abstract concepts into concrete, relatable experiences. When a medical trainee grapples with a challenging topic, the right metaphor can illuminate the path to understanding. We assert that the use of metaphor in medical teaching has a particular role in the creation of shared understanding to elucidate reflection and improve communication around complex and difficult topics.

While our focus is on the pedagogical utility of metaphor, we acknowledge a robust critical tradition examining how metaphors can skew understanding and reinforce problematic cultural norms (6–8). For instance, the pervasive “war” metaphor in medicine (7) can frame patients as battlegrounds and foster an adversarial relationship with disease. Similarly, Martin’s (6) seminal work reveals how gendered metaphors in biology can shape our very understanding of scientific processes. A mindful educator must therefore select metaphors not only for their explanatory power but also with an awareness of the implicit, and potentially limiting, conceptual baggage they carry (6, 9, 10).

The use of metaphors in medical communication draws from established principles in cognitive psychology, educational theory, and narrative medicine (11). Research demonstrates that metaphorical thinking engages multiple learning pathways, enhancing both understanding and retention (12, 13). The narrative medicine approach provides robust support for using storytelling and metaphor in medical practice, showing that medical knowledge is often best conveyed through relatable narratives (14, 15). Adult learning theory further supports this approach, emphasizing that adults learn best when new information connects to life experience and immediate relevance (16). This theoretical foundation applies equally to patient education and medical training, providing a unified framework for the dual application of metaphorical communication.

Colston argues that the creation of a cognitive duality using metaphor may be a hard-wired human trait, making metaphor the ideal way to share complex meaning across multiple domains (17). Bleakley’s book *Thinking With Metaphors In Medicine* explores the rise, fall, and rise again of metaphor in medicine. This was initially as an element of natural descriptive language, then discarded as imprecise and potentially harmful. A current renaissance of metaphor, however, argues it plays a vital role to make sense of emergent complexity in addition to addressing the challenges inherent in medical communication. In his chapter on the use of metaphor in medical education, Bleakley explores the potential role of metaphor in teaching clinical reasoning, ambiguity, and in exploring complex topics (18).

Used within medical education, metaphor also presents specific challenges that physicians should consider. Metaphors are contextual and the use of metaphors in intercultural, intergenerational, and interfaith conversations increases the chance of a missed metaphor. Additionally, neurodivergent individuals may process and understand metaphors differently than neurotypical individuals. In this paper, we will present a series of vignettes illustrating three practical uses of metaphor in medical education: (i) teaching clinical reasoning, (ii) opening up difficult conversations, and (iii) discussing professional identity formation. We also present a vignette of a missed metaphor, and a mnemonic, “SAFE,” describing an approach for medical educators to recognize and respond when a metaphor fails to deliver the appropriate message.

## 2 Part 1. Metaphor as a tool to teach clinical reasoning

Vignette: Dr. Alcott is an experienced family doctor working with a medical student, Meg. Meg has just seen a young patient who has booked a follow up visit to discuss panic attacks. Among other symptoms, the patient described having an unusual feeling in the chest during panic attacks. Meg has recently completed her cardiology rotation and knows that chest pain is a potentially dangerous symptom and proposes a full cardiac workup as a next step. Dr. Alcott wants to teach Meg about understanding symptoms in terms of the overall context of the patient and uses a metaphor to open the conversation: “A beeping smoke alarm means one thing if you are burning toast in the kitchen, and something entirely different if the alarm sounds in the middle of the night. In the first case, you wave a towel at it. In the second, you get out of the house and call the fire department. The beep is the same, but your response depends on the context.”

Most educators will be familiar with metaphors used early in medical education to simplify concepts, such as the “heart is a pump” metaphor. As training progresses, metaphor can be used to address more complex teaching topics. In the vignette above Dr. Alcott uses metaphor to help build a complex cognitive skill. The focus is on moving the learner from simple pattern recognition towards pattern recognition within a specific clinical context. This more complex metaphor focuses on how to interpret data and could open a discussion on probabilistic thinking, clinical reasoning, or resource allocation. It actively assists the learner to connect two different concepts: (i) certain symptoms are particularly concerning, and (ii) the context of the individual patient is important. Connecting these two concepts deepens understanding and potentially helps the learner to find meaning beyond the symptomatic medical “facts.” The meaning of symptoms depends on the context of the patient and although “chest pain” leads one swiftly down the coronary artery disease pathway, what “chest pain” means to this young patient needs to be unpacked beyond its literal meaning to appreciate the patient’s embodied experience.

Discussing the challenges of teaching clinical reasoning, Eva emphasizes the importance of recognizing the influence of context in clinical reasoning. Both setting, and recent exposure to similar cases, are identified as important factors. Eva suggests that medical educators must avoid the false dichotomy of analytical versus non-analytical thinking and expose learners to a variety of clinical reasoning strategies (13). Croskerry asserts that *thinking about thinking*, to attempt deeper understanding and awareness of our own cognitive processes, is the most important strategy to mitigate bias in clinical reasoning (19). Bleakley indicates that we must make medicine more imaginative, suggesting a role for metaphor in broadening our conception of how we perceive clinical reasoning (18).

In the example above, both preceptor and learner are influenced by their context. The preceptor is a family doctor, and the patient is a follow-up patient they already know. The learner has recently completed a cardiology rotation and is meeting a new patient for the first time. Their approaches to clinical reasoning differ: for Meg, the smoke alarm is sounding in the night, while for Dr. Alcott, the smoke alarm is due to a known cause, burnt toast. The metaphor is useful to explain Dr. Alcott’s approach to clinical reasoning in this case, but its greatest use is in opening a conversation about different forms of clinical reasoning and context-based approaches to consider not only the current reasoning strategy, but how multiple strategies might be applied to a case.

## 3 Part 2. Metaphor as an opening for difficult conversations

Vignette: Dr. Lee is an experienced emergency doctor working with a medical student, Jean. They have just completed a challenging case with a critically sick patient and had to give bad news to the patient’s family. Dr. Lee tries to open a discussion to debrief the case: “How are you feeling after that case?” Jean gives a closed off response: “I’m okay, I have seen some other critically sick patients before.” Dr. Lee changes the approach to opening the conversation, using a self-reflective metaphor: “I’m feeling very heavy after that case, like Atlas must have felt, carrying the weight of the world on his shoulders.” Jean pauses and then replies with a metaphor: “It was as if I was in a tornado, there were so many problems happening at once, I felt like I was spinning around and around, but could never fully catch up or hold onto any of the issues. It felt out of my control.”

There is abundant medical literature that focuses on managing difficult conversations between physicians and patients, and how to teach medical trainees these skills. In the medical education literature, the issue of difficult conversations is a part of the feedback literature. A variety of helpful feedback frameworks and techniques have been developed, but discussion of some topics remains challenging. Topics with significant emotional overlay, and topics that are difficult to articulate due to ambiguity and uncertainty, remain a challenge. Perhaps part of this challenge relates to the physician desire to use precise, certain, scientific language, and the interface of this desire with topics that are inherently imprecise and difficult to define. In these circumstances, the use of metaphor may offer a way to engage in these crucial conversations.

The lack of discourse around emotion within medical education has been identified as a challenge (20). Although physicians work within an environment rich in emotional context, dialogue about emotion is often lacking. The medical literature around managing emotionally difficult conversations between physicians and patients can inform similar conversations in medical education, and metaphor as a specific tool in these situations (21).

In the example above, Dr. Lee initially probes the learner’s emotional state directly and receives a closed off response. This will feel familiar to many medical educators, who might stop there, or move onto a more concrete matter in response. Instead, Dr. Lee broadens the context, moving from the learner’s emotion to the emotion surrounding the case, and makes a self-disclosure of their personal emotional state through metaphor. This serves to normalize the conversation and provide an example of metaphor as a technique to discuss emotion without the need for the precision of standard medical language.

The learner’s response in the above example may also open a door to discussions about additional important but challenging topics such as uncertainty, managing competing demands, or clinical courage. Although medicine is sometimes taught as if all cases end in proof and certainty, the actual practice of medicine at the front line demands a high tolerance for ambiguity. Metaphor offers a critical window into that ambiguity, providing a view into how we think as well as a reflection of what others, seeing the exact same situation, may experience in a completely different way (18). Indeed, Regehr, in speaking about medical education research, advises that the act of thinking in metaphors is what promotes the paradigm shift from simplicity towards complexity, and the tolerance of ambiguity (22).

While the metaphors of “Atlas” and the “tornado” successfully opened a dialogue about emotional weight and chaos, it is crucial to recognize that all metaphors simultaneously reveal and conceal (23). The “Atlas” metaphor might frame the emotional burden as a solitary, Herculean task, potentially foreclosing discussion about the value of shared responsibility or team support. The “tornado” metaphor captures a sense of uncontrollable chaos but may not adequately convey the moments of deliberate action that also occur in a crisis. The educator must approach the conversation with caution, recognizing that the metaphors proposed may require unpacking to explore their full implications and limitations.

## 4 Part 3. Metaphor as a tool to discuss professional identity formation

Vignette: Dr. Salinger is doing career counseling with a medical student, Holden, who is struggling to decide on a career path. They have discussed the nature of the work of different specialties many times, but this seems to cause Holden even more angst. Dr. Salinger introduces some metaphors to try to advance their conversation: “When you think of the kind of doctor you want to be, are you a mechanic who finds something broken and fixes it? Or are you a gardener who nurtures the plants and the soil and keeps watch for bad weather? Or are you a detective who finds subtle clues and uses them to put together a puzzle? or are you something else?” This pulls Holden back from the details about individual specialties and refocuses the conversation on his self-concept.

Professional identity formation describes the complex transformative process through which a professional integrates their preexisting skills, values, and behaviors, with those perceived to be required of their chosen profession (24, 25). Critical review of the professional identity formation literature in medical education indicates that the dominant modality, narrative reflection, may unintentionally support an individualistic view of professional identity, rather than a view of the professional as integrated within a larger culture (26). As a natural bridge between self and culture, metaphor may offer an alternative strategy for medical educators to teach and explore elements of professional identity with students.

The student in the above example will be familiar to many medical educators, struggling to plot their future course, and trying to reduce complex decisions about career paths to a simplified list of pros and cons. In this example, the wise teacher reframes the entire context of the conversation, using metaphor to ask the student how they see themself within, and as a part of a larger medical culture, rather than standing aside and apart from it. In this example, it is less important that the student identifies with one of the three metaphors that the teacher provides, and more important that they understand that they can describe their connection in a new way. The student can choose to make their own metaphor, deepening their understanding of themself, creating new meaning, and enabling them to take on the task of creating their own professional identity in a way that recognizes their own personal complexity.

The metaphors offered—“mechanic,” “gardener,” “detective”—each carry implicit worldviews. The “mechanic” implies a body-as-machine, where illness is a broken part to be fixed, potentially overlooking psychosocial dimensions (27). The “gardener” suggests a nurturing role but may inadvertently frame the patient as a passive entity to be cultivated. The “detective” focuses on solving a puzzle, which risks reducing the patient’s story to a set of clues. The power of this exercise lies not in identifying a single “correct” metaphor, but in sparking a metacognitive discussion about these very implications. By inviting the learner to create their own metaphor, the educator encourages a more nuanced, personal, and critically examined professional identity, one that acknowledges the complexity these simpler metaphors omit (28).

Varpio argues that most professional identity formation discourse is rooted in metaphors of *journey* or *fitting the mold* but that these metaphors do not capture the complexity or the actual process of professional identity formation. They *emphasize* the need for a new metaphor that does not constrain our thinking and propose a new metaphor of *professional identity formation as functional crafting* to emphasize process, individual variation, and creativity as fundamental parts of professional identity formation (28). Moreover, Bleakley also cautions about the limitations of old metaphors and asks us to be aware that traditional metaphors in medical education are often male gender coded (18). The need for new and creative metaphors to discuss professional identity formation is clear. In the example above, the preceptor does not suggest a single metaphor that will work for the learner but rather offers a few potential metaphors and invites the learner to create their own, which may have more meaning. The use of metaphors in this discussion may allow the learner to conceptually envision different versions of their future self, metaphorically trying on different coats, seeing which one feels like it may fit, before choosing to wear it more permanently.

## 5 Part 4. When metaphors miss: using the SAFE strategy

Vignette: Dr. Alcott has had previous success discussing the clinical reasoning using metaphor and so tries it with a new student, Beth, choosing a well-known metaphor: when you hear hoofbeats think of hoofbeats, not zebras. Beth looks puzzled for a moment and then says “I do not understand how hoofbeats would tell you anything about whether a horse of a zebra is making the noise, they are at least equally likely.” Realizing that the metaphor fell off the mark, Dr. Alcott shifts into the SAFE strategy, “I think my metaphor has missed the mark. Can you tell me more about why you would say that?” Beth replies “Well Dr. Alcott, where I grew up in South Africa, a hoofbeat could be a zebra or a horse, so the sound does not really help you figure it out.” Dr. Alcott acknowledges the cultural and geographical miss and explains in simple terms “thank you for sharing that, knowing that now, I would have chosen a different metaphor. My intention was to illustrate how common symptoms are usually linked to common causes.”

There are scenarios where metaphors can miss the mark, potentially create more confusion or ambiguity, or even cause offense or harm. Bleakley argues that metaphor is not entirely in the mind or entirely within the culture but acts as a bridge between the two (18). The role of culture must be considered when using metaphor. Conceptual metaphors map information between two different contextual domains. Although some concepts may be universal, the majority are culturally specific or culturally informed. Where contextual domains differ, potential for loss of meaning, misunderstanding, and harm exists (29). Culture should be understood broadly in this context. Interfaith, international, intergenerational, and other forms of communication between individuals who may use language and conceptual ideas differently has the potential for intercultural misunderstanding.

The metaphor presented in the vignette is one of the most used metaphors in medicine, but it too has both baggage and potential for misunderstanding. While it may feel safe to use such a well-worn metaphor, the teacher must not forget that the metaphor itself derives from a specific culture and context. The “hoofbeats/horses/zebras” metaphor is rooted in a western worldview where horses are ordinary, and zebras are rare. This could be confusing for a learner from a non-western background and could also be taken to mean that the diseases usually observed in the western world (“horses”) deserve more consideration than the diseases more common in non-western populations (“zebras”). While metaphor may create meaning for some learners, there is potential for confusion, offense, or even harm when a metaphor misses the mark.

There is also evidence that some neurodivergent individuals process and understand metaphors differently than neurotypical individuals. Some neurodiverse individuals may take longer to process metaphor, some may find metaphor confusing rather than enlightening, while others may process metaphor normally (30–32). A neurodiverse learner who thinks literally might ask *“Why are we talking about horses and zebras? We’re not in veterinary school.”* Frequently, neurodiverse medical trainees do not disclose this status (33) and so medical educators need to consider this as a potential reason why a metaphor may fail to deliver the intended message, including with trainees who they do not know to be neurodivergent.

Some metaphors may inadvertently have built in harmful implications. Fatehi uses the example of how the *wear on a tire* metaphor to describe osteoarthritis may inadvertently implicate physical activity as the cause of disease (34). Given one of the primary first management recommendations for osteoarthritis is movement and exercise, to implicate physical activity as a culprit metaphorically may impact patient comprehension and subsequently their compliance. Medical educators need to be cautious in their selection of both when to use metaphor, and the metaphors themselves, and be prepared to respond if a metaphor misses the mark.

**Figure 1:**
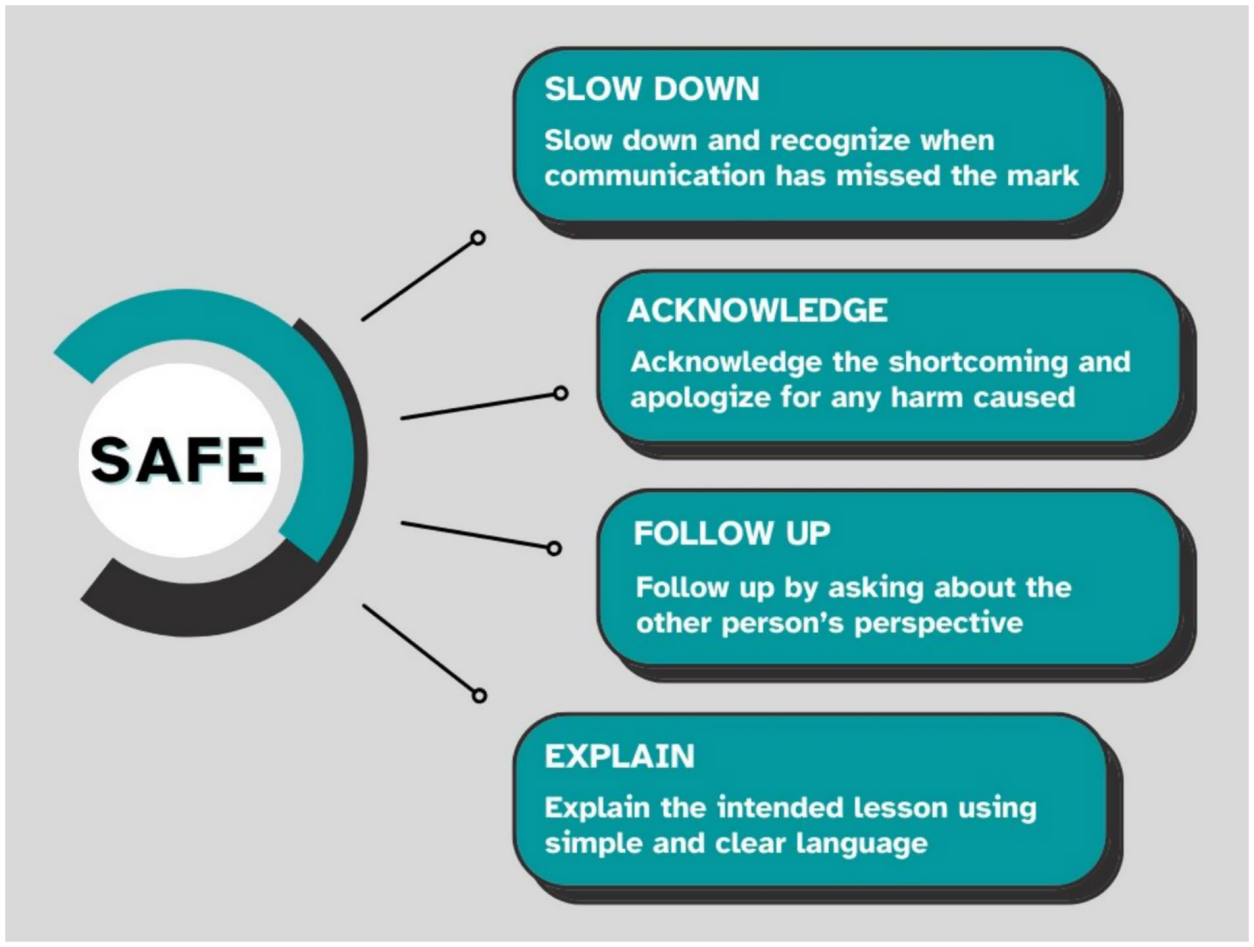
The SAFE strategy.

The SAFE strategy (Figure 1) outlines a simple process for recognizing and addressing lapses in communication. While the principles are broadly applicable to any communicative misstep, we think that they are particularly important for educators who use metaphor, because of the specific risks that metaphor can introduce into the learning environment. Step 1 involves slowing down and recognizing when communication has fallen short in messaging. An educator using metaphor should be aware of reactions or body language that indicate that a metaphor fails to deliver the message. When recognized the teacher should slow down and address the issue, rather than continuing to teach.

Step 2 involves acknowledging the miss and apologizing if harm was caused. While educators do not intend to harm, students can be harmed, as demonstrated in the vignette when what the teacher believes is a simple context overlaps with important cultural or spiritual context from the learner’s perspective.

Step 3 involves following up to understand the other person’s perspective. This allows an understanding of the learner’s context and ensures that the mistake will not be repeated.

Step 4 involves explaining the intended lesson in clear language. This serves the purpose of clarifying the teacher’s intent and ensuring that the educational aim is not lost.

The bilateral exchange of the selection and meaning of metaphors is described as a technique to promote intercultural understanding (35), but this is recognized as an advanced technique, and while potentially of interest, it is time consuming and difficult for clinical educators to use in a typical clinical education scenario. We have not included this in the SAFE mnemonic, but this may present an additional next step for interested educators. Ultimately, metaphors can enhance a learner’s capacity to relate to a patient’s story but can also confuse and harm learners in the wrong context. Therefore, it is important that the teacher discern when a metaphor may prove unfruitful and be prepared to manage the misunderstanding.

## 6 Conclusion

The use of metaphor has experienced a rise, fall, and recurrent rise in clinical medicine and medical education over time. The current renaissance of metaphor offers an opportunity to be more intentional in its use, applying it in situations where it can be most impactful. We describe three practical areas where medical educators might employ a metaphor to teach clinical reasoning, to discuss difficult topics, and enhance professional identity formation. Metaphor must be used cautiously; some are outdated, and many medical metaphors are male gender coded. Educators must be aware of challenges when using metaphor across cultures, and with neurodiverse individuals, and be ready to respond when a metaphor misses the mark using the SAFE strategy.

## Data Availability

The original contributions presented in the study are included in the article/supplementary material, further inquiries can be directed to the corresponding authors.
